# Impact of Supplementary Amino Acids, Micronutrients, and Overall Diet on Glutathione Homeostasis

**DOI:** 10.3390/nu11051056

**Published:** 2019-05-11

**Authors:** Rebecca L. Gould, Robert Pazdro

**Affiliations:** Department of Foods and Nutrition, University of Georgia, Athens, GA 30602, USA; rebecca.gould@uga.edu

**Keywords:** Glutathione, amino acids, micronutrients, supplementation, homeostasis

## Abstract

Glutathione (GSH) is a critical endogenous antioxidant found in all eukaryotic cells. Higher GSH concentrations protect against cellular damage, tissue degeneration, and disease progression in various models, so there is considerable interest in developing interventions that augment GSH biosynthesis. Oral GSH supplementation is not the most efficient option due to the enzymatic degradation of ingested GSH within the intestine by γ-glutamyltransferase, but supplementation of its component amino acids—cysteine, glycine, and glutamate—enhances tissue GSH synthesis. Furthermore, supplementation with some non-precursor amino acids and micronutrients appears to influence the redox status of GSH and related antioxidants, such as vitamins C and E, lowering systemic oxidative stress and slowing the rate of tissue deterioration. In this review, the effects of oral supplementation of amino acids and micronutrients on GSH metabolism are evaluated. And since specific dietary patterns and diets are being prescribed as first-line therapeutics for conditions such as hypertension and diabetes, the impact of overall diets on GSH homeostasis is also assessed.

## 1. Introduction

Glutathione (GSH) is a low molecular weight, water-soluble thiol that is present in all eukaryotic cells [1]. It is synthesized from the amino acids glutamate, cysteine, and glycine, produced exclusively in the cytosol [2], and transported to other subcellular organelles, including mitochondria [3], endoplasmic reticulum [4], and the nucleus [5]. Throughout these locations, glutathione is most commonly found in its reduced form [6], GSH, which is consumed during redox reactions to produce the oxidized form, GSSG. GSH is the most abundant non-protein thiol in mammals [7], playing important roles in peroxide detoxification [8], recycling of vitamins C and E [2,9,10], cysteine storage [11,12], and other biochemical reactions. Oxidative stress accelerates the rate of GSH conversion to GSSG, thereby decreasing the ratio GSH/GSSG; such a shift has major impacts on cellular signaling [13], thiol disulfide exchange reactions [14], and cell proliferation [15]. Alterations in GSH homeostasis have been associated with protein energy malnutrition (PEM) [16], cancer [17,18], Alzheimer’s and Parkinson’s disease [16,18,19], HIV [19] and AIDS [18], liver and heart disease [18], aging [17,19], diabetes mellitus (DM) [18,20], and obesity [21].

Due to the strong cytoprotective effects of GSH, it could be a therapeutic option to maintain cellular integrity and slow tissue degeneration in various diseases. Researchers have tested the impact of administering GSH through several routes, including intravenously [22], intranasally [23], orally [24,25,26], sublingually [27], via inhalation [28], and transdermally [29]. Given the invasiveness and financial burden of many of these routes, oral administration is considered most convenient [27]. Yet, the efficacy of oral GSH against oxidative stress is still controversial due to several unresolved issues [30]. Notably, oral GSH supplementation had no effect on oxidative stress biomarkers or GSH concentrations in red blood cells (RBC) isolated from healthy human subjects [24,31], a finding that may be attributable to GSH digestion. The primary site of dietary GSH digestion is the upper jejunum [32], where the enzyme γ-glutamyltransferase (GGT) breaks down GSH to its constituent amino acids [24]. GGT is also expressed in the liver [33], and these combined actions limit the amount of dietary GSH that enters the circulation. Furthermore, most cells simply cannot absorb intact GSH, instead requiring it to be first broken down by GGT into its constituent amino acids [34]. Because of these issues, researchers have instead turned to supplementation of individual GSH precursors to improve GSH status. Several amino acids intersect with the GSH pathway, and altering the concentrations of those amino acids, either directly or indirectly, can modify cellular GSH homeostasis.

This review presents the effect of oral supplementation of individual amino acids on GSH homeostasis in mammals with emphasis on clinical studies. Then, we highlight particular micronutrient supplements that have been found to improve GSH status by generally enhancing cellular antioxidant systems. Lastly, since specific dietary patterns are commonly being prescribed as first-line therapeutics for certain chronic diseases, this review will also briefly assess the impact of these overall diets on GSH homeostasis.

## 2. Effect of Individual Amino Acid Supplementation

### 2.1. L-Glutamine

L-Glutamine is the most abundant free amino acid in the body [35]. Through normal metabolic pathways, glutamine is converted to glutamate, which is used in the intracellular synthesis of GSH. It has been postulated that through direct oral supplementation of glutamine, GSH levels would consequently increase.

When healthy human volunteers were supplemented with oral glutamine at 0.3 g/kg/day for 10 days, both glutamine and glutamate concentrations increased in plasma, but blood GSH concentrations unexpectedly decreased [35]. This effect may be attributable to competition between amino acids for absorption. Glutamate shares a saturable transporter with cystine [36], the oxidized form of cysteine, the rate-limiting amino acid in GSH biosynthesis [2]. Excess extracellular glutamate could therefore reduce cysteine availability [37], reducing cysteine transport into RBC and other cells and driving a subsequent decrease in GSH synthesis [35].

It has been proposed that glutamine most effectively increases GSH levels during times of GSH depletion [35]. Therefore, glutamine supplementation should be tested in patients with low GSH status. For instance, HIV patients typically present with low blood concentrations of GSH and its amino acid precursors, and consequently, this population is ideal for testing GSH-boosting interventions [38]. When 12 HIV^+^ patients were supplemented with glutamine (20 g/day) for 7 days, plasma GSH, glutamine, and glycine levels were normalized [38]. However, plasma cysteine and glutamic acid were not restored to normal levels, nor did it significantly alter the plasma GSSG/GSH ratio [38]. 

Glutamine supplementation may not be as effective in patients in the intensive care unit (ICU) [39,40] or with sickle cell disease [41] {Morris, 2010 #1278}. When 120 ICU patients with either peritonitis or trauma were supplemented with glutamine (45 g/day) for 5 days, blood GSH levels increased in both the supplemented and non-supplemented patients at similar rates, indicating that glutamine replacement may not be the causative agent [40]. Similarly, when 5 sickle cell disease patients were supplemented with glutamine (10 g mixed with Gatorade), RBC GSH appeared to trend upward, but results were not statistically significant [41] {Morris, 2010 #1278}. However, glutamine and arginine availability increased, so it is plausible that effects on GSH concentrations may have reached significance with further supplementation [41] {Morris, 2010 #1278}.

Rodent studies support the notion that GSH levels increase with glutamine supplementation [42,43,44,45]. Paraquat, a toxic herbicide, was used to induce oxidative stress in rats, and when those rats were orally supplemented with glutamine, plasma, but not renal and hepatic GSH, increased [42]. When malnourished rats in inflammatory shock were supplemented with glutamine, gut, but not hepatic, GSH levels were restored [45]. Furthermore, in rats with breast cancer, oral glutamine supplementation increased blood, breast tissue, and gut mucosa GSH levels [43]. However, glutamine supplementation had no effect on diabetic rats [46]. Studies in rats enduring high-intensity resistance [47], long-duration exercise [48], and endotoxemia [49] have found that glutamine may be more beneficial when paired with alanine, an area of research that needs to be tested in human subjects.

It must be noted that long-term glutamine supplementation should not exceed >40 g/day due to its negative effects on biochemical pathways [50]. Glutamine not only competes for absorption against other amino acids such as cystine, but also impairs endogenous glutamate synthesis [50]. Furthermore, glutamine supplementation can increase ammonia production while also impairing ammonia transport and detoxification [50]. Overall, glutamine supplementation appears to be beneficial in HIV^+^ individuals, but not in ICU patients or those with sickle-cell disease. It is crucial that future research efforts expand beyond RBC, as RBC lack mitochondria, producing a unique biochemical milieu that may not fully represent other cell types [35].

### 2.2. L-Glycine

Glycine is one of three amino acids used to form GSH [51], so it has been postulated that its oral supplementation may stimulate GSH biosynthesis [52]. In one study, fourteen-day-old piglets were supplemented with glycine in a liquid milk replacer [52]. Plasma GSH concentrations increased significantly while the respective ratio of GSSG/GSH decreased in a dose-dependent fashion [52]. To determine whether glycine would have a similar effect in a model with low GSH status, many studies have employed animal models with metabolic syndrome, which causes oxidative stress and vascular complications [53]. Researchers proposed that supplementing sucrose-fed rats with glycine would increase GSH content in the aorta, providing vascular tissue with crucial protection against reactive oxygen species (ROS) and endothelial dysfunction [53]. In fact, supplementation of glycine for 4 weeks resulted in an 85% increase in aortic GSH, 64% decrease in aortic GSSG, and an overall significantly higher GSH/GSSG ratio [53], with a concomitant increase in expression levels of GSH biosynthetic enzymes. In control rats, GSH increased while GSSG did not significantly change [53]. In a related study, sucrose-fed rats were supplemented with glycine through their drinking water, a treatment that increased GSH levels and GSH/GSSG ratios in the liver while hepatic protein carbonyls, a marker of oxidative stress, decreased [54].

Glycine seems to improve GSH concentrations in animals with higher levels of oxidative stress. When supplemented in the diet, glycine may exert a beneficial effect via promotion of GSH biosynthesis and attenuation of oxidation, which in turn protects against endothelial and vascular dysfunction. There is evidence that glycine supplementation could be even more effective when orally supplemented with cysteine in older HIV-infected individuals [55]. The role of glycine supplementation in improving GSH status warrants further research given its broad, beneficial influence on the GSH pathway.

### 2.3. Cysteine Pro-Drugs

*L*-cysteine is the rate limiting amino acid in de novo synthesis of GSH [2], so naturally, cysteine supplementation should be investigated as a possible approach to improve GSH status and counter disease-associated oxidative stress. Yet according to studies on this topic, direct administration of cysteine is limited by spontaneous oxidation to its corresponding disulfide cystine [56], significant toxicity [57,58,59,60,61,62], and mutagenicity risks [63,64]. Because of these issues, focus has shifted from cysteine to its precursors, which may provide an alternate avenue to raising GSH concentrations by increasing the available cysteine pool.

The efficacy of cysteine precursors *N*-acetylcysteine (NAC), *S*-Adenosyl-*L*-methionine (AdoMet), and *L*-2-oxothiazolidine-4-carboxylate (OTC) supplementation on GSH levels are evaluated in the next sections. Methionine is also a cysteine prodrug that increases RBC GSH levels when supplemented orally [65], but supplementing with methionine is not recommended due to the increased risk of raising homocysteine levels [66,67], so it will not be discussed in this review.

#### 2.3.1. N-Acetylcysteine (NAC)

NAC is a cysteine precursor that, when administered orally, increases GSH concentrations in individuals with GSH deficiency caused by infections, genetic defects, or metabolic disorders [68]. Orally administered NAC is readily absorbed in the stomach and gut [69] and then converted to cysteine in the liver [70], where it is used locally for GSH synthesis or transported throughout the body to individual tissues [71]. Pharmokinetics studies have revealed that NAC is almost entirely metabolized by the liver and kidneys [70], and as a result, NAC is nearly undetectable in plasma [72]. Therefore, measuring NAC in plasma is not representative of NAC status [72].

In a study by De Rosa et al., HIV-infected individuals were orally supplemented with 8000 mg NAC daily for 8 months, and whole blood GSH levels significantly increased with no major adverse effects [73]. An additional study found that when 12 HIV^+^ patients were orally supplemented with NAC (1 g/day), plasma GSH, cysteine, homocysteine, taurine, methionine, and glutamine concentrations increased significantly [38]. In a separate treatment group, glutamine was supplemented (20 g/day) daily, and results were contrasted against those of NAC. The effects of NAC were greatest, achieving an almost two-fold increase in plasma GSH levels [38]. In addition, NAC supplementation was found to benefit individuals with low resting GSH levels [74], and may be even more effective when supplemented with glycine [75].

Overall, orally administered NAC is an effective way to replenish GSH in HIV-infected patients, but more research is needed to better understand the effect of NAC supplementation on GSH levels in healthy individuals. Despite its potential benefits, NAC is limited by potentially harmful side effects, such as nausea or gastric distress, but investigators hypothesize that the intestinal side effects found in the trial may have been due to the ingestion of the excipient, which contained lactose [68].

#### 2.3.2. S-Adenosyl-L-Methionine (AdoMet)

AdoMet is a precursor to the transsulfuration pathway, the metabolic pathway responsible for condensing homocysteine to form cystathionine, which is then converted to cysteine [76]. AdoMet is decreased in individuals with liver disease, resulting in GSH depletion [77,78,79]. Patients with alcoholic liver disease (ALD) or non-alcoholic liver disease (NAFLD) were found to exhibit low hepatic GSH levels, which were replenished after oral AdoMet treatment (1.2 g/day for 6 months) [77]. Rodent studies have supported this finding, concluding that AdoMet supplementation is effective at attenuating liver injury by increasing GSH levels [80,81,82,83,84].

In baboons, supplementation of oral AdoMet resulted in significant hepatic uptake of the molecule, and eventually, restored GSH levels [85]. These results are important, as non-human primates model liver disease pathology better than rodents, which are less likely to develop ALD [86]. Impaired methionine metabolism is a major contributor to hepatic GSH dysfunction in liver disease patients, and rodents naturally express higher levels of betaine-homocysteine methyltransferase, an enzyme which maintains proper hepatic methionine function [86]. Therefore, AdoMet supplementation may not alter GSH status in rodents who already have innate compensatory mechanisms for overcoming methionine dysfunction [86].

AdoMet supplementation does not appear to have a significant effect on homocysteine concentrations in healthy individuals [87], so supplementation of this amino acid may be a viable source of increasing GSH levels without deleterious consequences of increased homocysteine.

#### 2.3.3. L-2-Oxothiazolidine-4-Carboxylate (OTC)

OTC is converted to cysteine through 5-oxoprolinase in cells [19] and has been used to elevate GSH concentrations in animal models [88,89,90,91,92,93] and humans [94,95]. Much like the other cysteine prodrugs, OTC supplementation has been evaluated for its effect on GSH homeostasis in populations with low GSH statuses. In a study conducted on rats with liver injury due to alcohol or a high-fat diet (HFD), oral supplementation of OTC for 1 month resulted in increased circulating GSH levels [88]. Furthermore, OTC protected against liver injury [88]. In another study, PEM rats receiving daily OTC significantly increased GSH in the lungs, but OTC was not as effective as protein repletion in restoring blood GSH levels [96]. In healthy humans, oral OTC supplementation was found to increase the lymphatic GSH concentration 2 to 3 h after ingestion, but plasma GSH remained unaffected [94].

Studies suggest that OTC may be a safe and viable option for elevating GSH concentrations, but some limitations remain unaddressed. A study by Nishina, Ohta, and Obuka (1987) found that intraperitoneal delivery of OTC increased hepatic GSH concentrations in guinea pigs, but decreased renal GSH levels [97]. It was suggested that hepatic OTC was used to form cysteine for GSH synthesis, whereas in the kidney, OTC promoted GSH turnover instead [97]. New efforts should focus on whether orally supplemented OTC causes a similar effect, so that treatments can be tailored accordingly.

### 2.4. L-Serine

The transsulfuration pathway is a major source of sulfur for GSH formation [98]. In the pathway, cystathionine β-synthase, along with vitamin B_6_, condenses homocysteine and serine to ultimately form cysteine [98]. Presently, research on oral serine supplementation on GSH homeostasis is limited and mostly confined to rodent models with high levels of oxidative stress.

In mice fed a HFD, supplementation of serine in drinking water significantly decreased hepatic ROS compared to the control group [99]. Serine also increased hepatic GSH concentrations, GSH/GSSG ratios, and expression of GSH-related enzymes glutathione reductase (GR), glutathione peroxidase (GPx), and glutathione S-transferase [99]. Furthermore, serine-supplemented mice exhibited lower hypermethylation rates in the promoter region of GSH synthesis-related genes [99]. Serine had similar effects on hepatic GSH metabolism and intestinal GPx activities in diquat-treated mice [100] and early-weaned piglets [101], respectively. In addition, serine supplementation increased hepatic GSH levels in mice and rats with ALD [102]. To evaluate the effects of serine supplementation at old age, researchers treated 18-month-old C57BL/6J male mice with 0.1, 0.2, or 0.5% w/v serine in their drinking water and found that the highest dosage increased serum GSH levels [103]. Moreover, long-term serine supplementation was associated with reduced food intake and age-related weight gain, stemming from a possible interaction between serine and hunger signaling pathways, as well as activation of the “longevity gene,” *Sirt1* [103].

In experimental models of oxidative stress, serine appears to conserve tissue GSH levels and attenuate oxidative damage. But more research is needed to better define mechanisms by which serine protects GSH pools and acts on *Sirt1* and other age-related pathways.

### 2.5. Taurine

Cysteine is a necessary metabolic precursor for taurine synthesis, so it has been postulated that taurine supplementation would spare cysteine for eventual incorporation into GSH [104]. In research studies on this topic, animals were supplemented with taurine intraperitoneally [104], via drinking water [105,106,107,108,109,110], or intragastric intubation [111]. When investigators supplemented diabetic rats with taurine, lens GSSG and GSSG/GSH ratio levels decreased while GSH levels were not affected [112]. In rabbits on a high-cholesterol diet, taurine failed to affect hepatic GSH levels [113]. However, taurine supplementation normalized GSH and GSSG levels in iron-overloaded mice [110] and increased renal and hepatic GSH levels in rats with hypothyroidism [109].

Effects of taurine on the enzymes GPx and GR have been evaluated in both rodent and human studies. Taurine supplementation recovered hepatic GR activity in diabetic rats [114], whereas hepatic GPx levels remained unaltered by taurine supplementation in type I and type II diabetic mice [105]. However, when young adults were orally supplemented with taurine (50 mg/kg for 2 weeks) after performing eccentric exercise, RBC GPx levels were elevated [115].

Overall, these results point to a limited impact of taurine on GSH, implying that other amino acid or micronutrient supplements may be a more effective option at targeting and raising GSH concentrations.

## 3. Effects of Micronutrient Supplementation

### 3.1. α-Tocopherol (Vitamin E)

Fat-soluble vitamins (A, D, E, and K) have been evaluated for their potential effects on GSH homeostasis. These vitamins exhibit antioxidant properties, and there is evidence that status of vitamin A [116,117], vitamin D [118,119,120], and vitamin K [121] may affect GSH levels; supplementation has also been associated with adverse effects [117]. Given the nature of this review, we will focus on the fat-soluble vitamin that directly affects GSH concentrations through its antioxidant capabilities: vitamin E.

Vitamin E is a lipid-soluble antioxidant that protects the cell membrane against oxidation and supports other antioxidant reactions, including those involving GSH [122]. Studies suggest that vitamin E supplementation may prevent GSH oxidation and ultimately increase its levels in tissues. To test this concept, rats were fed a normal or vitamin E-supplemented diet for 15 days and subjected to a single dose of ethanol [122]. Vitamin E increased GSH levels in the cerebral cortex and cerebellum, while maintaining GSH status in ethanol-treated rats [122]. Furthermore, vitamin E supplementation increased renal GSH levels in exercise-stressed rats [123], increased both RBC GPx and renal GSH concentrations in healthy rats [124], and when supplemented with Se, increased rat hepatic GPx expression [124]. Yang et al. stated that vitamin E supplementation of 25–250 IU/kg is the optimal dosage in rats to maintain optimal GPx activity [125].

The beneficial impact of vitamin E on GSH translates to clinical studies as well [53,126,127,128]. Type 1 diabetic children initially exhibited lower RBC GSH concentrations compared to healthy subjects [129], and when those children were orally supplemented with vitamin E (100 IU/day) for 3 months, RBC GSH levels increased while lipid peroxidation and HbA_1c_ levels decreased [129]; the results are consistent with past animal studies on this topic [130]. In addition, vitamin E supplementation (1000 IU/day for 10 days) increased RBC GSH levels in healthy volunteers [127]. However, when vitamin E-deficient thalassemic children were supplemented with vitamin E for (200 mg/day) for 4-8 weeks, RBC GPx activity decreased [131] and supplementation of 400 mg/day for 2 months failed to raise plasma GSH levels in elderly cardiovascular patients [132].

Based on these rodent and clinical studies, it appears that vitamin E, when taken in safe doses, has the potential to increase RBC and brain GSH concentrations.

### 3.2. Pyridoxine (Vitamin B_6_)

Pyridoxal 5′-phosphate (PLP), the biologically active form of vitamin B_6_, is a coenzyme in the conversion of homocysteine to cysteine, which supports GSH biosynthesis [133]. PLP is also a powerful antioxidant that has an indirect role in GSH homeostasis via sequestering free radical species to spare cellular GSH concentrations [134,135].

Homocysteinemia and chromium toxicity are high stress environments wherein vitamin B_6_ is especially critical for maintaining GSH pools [135,136,137]. Oral vitamin B_6_ supplementation increased renal and hepatic GSH levels in rats with chromium-induced toxicity [136,137] and increased hepatic GSH levels in homocysteinemic rats, despite the fact that homocysteine levels remained high [135]. Conversely, vitamin B_6_ supplementation had no effect on hepatic GSH levels in control rats, with or without supplementation [135], nor did it alter GPx levels in homocysteinemic rats [133]. Unexpectedly, vitamin B_6_ supplementation increased blood GPx levels of control rats, indicating that the vitamin may directly stimulate the GSH redox system under normal conditions [138].

Vitamin B_6_ also affects GSH status of human patients. Hepatocellular carcinoma (HCC) patients exhibit high levels of oxidative stress, and when 33 HCC patients—who had recently undergone a tumor resection—were supplemented with vitamin B_6_ (50 mg/day) for 3 months, plasma GSH/GSSG ratios and GR activity unexpectedly decreased while GSSG and GPx levels did not change [139]. The authors hypothesized that the decline in GSH came from redistribution of GSH molecules from plasma to RBC [139].

The impact of vitamin B_6_ supplementation with other vitamins has been also evaluated [140,141]. Combined vitamin B_6_ (200 mg/day) and folic acid (15 mg/day) supplementation for 1 month increased RBC and whole blood GSH levels in both hemodialysis patients and healthy patients [141]. Similarly, Parkinson’s disease patients supplemented with folic acid and vitamins B_12_ and B_6_ had improved GSH status [140].

At this time, recommendations on B_6_ supplementation cannot be made due to the limited clinical research and conflicting results on the effect of vitamin B_6_ supplementation on GSH concentrations. Future research on vitamin B_6_ supplementation is warranted given the possibility of beneficial effects on GSH status.

### 3.3. Ascorbate (Vitamin C)

Ascorbate, a potent free radical scavenger, and GSH are biochemically intertwined [142,143]. GSH actively recycles oxidized dehydroascorbate back to ascorbate [144], and GSH deficiency is accompanied by low tissue levels of ascorbate [143]. It has also been predicted that increasing ascorbate levels ultimately spares GSH pools [145].

Rodent models have clarified the role of ascorbate in GSH metabolism. Ascorbate normalized hepatic GSH levels in homocysteinemic rats, despite the fact that serum homocysteine levels remained high [135]. When aged rats were supplemented with ascorbate, pulmonary GSH increased [146], and total glutathione and GSH/GSSG ratios increased in chronically loaded muscles [9]. But in weanling rats supplemented with ascorbate in high doses for 4 months, RBC GSH concentrations actually decreased [147]. Moreover, there was a significant increase in GPx activity in RBC and plasma [147]. Similarly, heart GPx and GSSG levels increased after 5 weeks of high ascorbate supplementation in guinea pigs [148]. Investigators attributed these negative results to peroxidative stress induced by the high ascorbate doses [147].

Healthy adults were supplemented with ascorbate to better understand the vitamin’s effects on GSH homeostasis. Subjects were supplemented with a placebo for 1 week, 500 mg/kg/day for 2 weeks, 2000 mg/day for 2 weeks, then placed on a placebo-controlled withdraw week [149]. RBC GSH levels significantly increased by 50% after weeks 2–3 (500 mg/day) but were not correlated with RBC ascorbate concentrations [149]. There was no significant difference in RBC GSH concentrations between the 500 mg/day group and the 2000 mg/day group [149]. In addition, RBC GSH concentrations did not significantly change after the placebo-controlled withdraw period, indicating that ascorbate supplementation may be a feasible way to improve and maintain reduced GSH stores in RBC [149]. Furthermore, ascorbate supplementation (500 or 1000 mg/day) was found to increase lymphocyte GSH levels in healthy adults [150].

Ascorbate has also been combined with vitamin E since the two vitamins have an additive effect against oxidative stress [9]. When ascorbate was supplemented (500 mg/day) with vitamin E (400 IU/day) for 2 months in cardiovascular disease patients, GPx levels were significantly higher than those without supplementation [151]. Moreover, supplementation of the two vitamins increased GPx activity in women consuming oral contraceptives, a population that has been found to have decreased antioxidant status [152].

Based on this evidence, ascorbate, if used in safe and tolerable doses, appears to attenuate GSH depletion under stress conditions and promote an increased antioxidant status.

### 3.4. Selenium (Se)

In the enzymatic reduction of hydrogen peroxide to water, GSH is utilized by GPx, a selenoprotein that requires Se for proper functioning [153,154]. Researchers have predicted that increasing Se would increase cellular GPx expression and lower oxidative damage in tissues. Indeed, oral Se supplementation has been shown to improve GSH status in rodents [124,155,156,157]. Clinical interventions have been conducted to determine whether Se exerts a comparable effect in humans. Supplementing women with Se (200 mcg/day) for 4 weeks increased platelet, whole blood, plasma, and liver GPx expression levels [158]. In another study, 45 patients with chronic kidney disease (CKD) were supplemented with 200 mcg of Se for 3 months, and their RBC GPx activities increased [159]. The same dosage was used in 53 chronic renal failure patients, and Se increased plasma GPx among those in the early stages of the disease, but not in end stage renal disease patients [160]. Zachara et al. evaluated the effect of Se supplementation in patients undergoing hemodialysis (HD). When dialyzed uremic patients were supplemented with 300 mcg Se after each hemodialysis session (3 times/week) for 3 months, GSH levels did not change, but GPx activity increased after only 1 month of supplementation [161]. Overall, the relationship between Se status and GPx activity has been inconsistent, with some studies concluding that Se supplementation has no effect on GPx activity in healthy individuals [162,163,164] or in those with mucopolysaccharidosis [165], while others found beneficial effects [166,167,168]. Such conflicting outcomes could be a result of oral Se supplementation acting in a tissue-specific manner on GSH homeostasis [169].

Further research is needed to better define the impact of Se on GSH concentrations, given its proven ability to increase GPx protein levels. Although Se could be a viable option to increase GPx activity in individuals with high oxidative stress, Se supplementation may be most effective in combination with another therapeutic known to directly increase GSH. After all, without GSH, an increase in GPx may not induce significant health effects.

### 3.5. Magnesium (Mg)

Mg is an important mineral whose status has been linked to oxidative damage [170]. When diabetic rats were orally supplemented with Mg, blood concentrations of total glutathione and GSH increased [171]. However, since whole blood was analyzed, it is possible that the effect was driven by increased GSH secretion from cells [171]. In addition, women of unexplained infertility or early miscarriage were found to have abnormal RBC-Mg levels, and when these women were supplemented orally with Mg (600 mg/day) for 4 months, those who still had abnormal RBC-Mg levels also had significantly lower RBC GPx levels [172]. After 2 additional months of Mg supplementation, RBC-Mg levels and GPx concentrations were normalized in all women [172]. Furthermore, when atopic asthmatic children (4 to 16-year-olds) were orally supplemented with Mg for 3 months, blood GSH levels increased while blood GSSG remained unaffected [173]. But when pregnant women (24–28 weeks) with gestational diabetes were supplemented with 250 mg Mg/day for 6 weeks, plasma GSH concentrations did not significantly change [174].

Based on these clinical trials, it is possible that Mg supplementation may provide antioxidant protection. Importantly, more research is needed to identify the optimal dose of Mg supplementation to boost GSH metabolism.

## 4. Overall Diet Effects

### 4.1. The Mediterranean Diet (MedDiet)

The Mediterranean diet (MedDiet) emphasizes high intakes of extra virgin olive oil, vegetables, fruits, cereals, nuts, pulses and legumes, and moderate intakes of fish, dairy, and red wine, while limiting intakes of eggs and sweet foods [175]. There is typically no specific serving size recommended, but instead, emphasis is placed on frequency of food consumption [175]. The diet’s effect on oxidative stress levels has been a subject of investigation, given its high content of Se, essential fatty acids, fiber, and antioxidants [176]. Using data from the Twins Heart Study, researchers explored a potential association between the MedDiet and GSH levels [177]. Adherence to the MedDiet, based on Mediterranean Diet Scores (MDS), showed an inverse association with plasma GSSG, resulting in higher GSH/GSSG ratios, independent of familial and genetic factors [177]. In addition, the MedDiet was found to enhance GPx activity in patients with atrial fibrillation [178] and increase mammary gland GSH levels in monkeys that were on the diet for 31 months [179]. In a cross-sectional study of healthy working men and women, MDS were positively associated with plasma GSH levels while similar diets, such as the Alternative Healthy Eating Index and the Dietary Approaches to Stop Hypertension (DASH) diet, were not [180]. These conclusions were independent of BMI, an important factor given the strong correlation between fat accumulation and increased systemic oxidative stress [181].

Efforts to understand the relationship between the MedDiet and GSH levels should now be expanded. For instance, new efforts may be conducted in pediatric populations to identify any protective antioxidant mechanisms that may be enhanced when beginning the diet early in life.

### 4.2. The DASH Diet

The DASH diet was developed in the 1990s and rose in popularity once the National Institutes of Health began supporting research efforts to identify dietary interventions that were efficacious against hypertension [182]. The DASH diet was found to decrease systolic blood pressure by 6–11 mm Hg in both hypertensive and normotensive individuals and is therefore considered a first-line therapy for hypertension today [182]. The DASH diet includes the following servings of foods per day: 5 fruits and vegetables, 7 carbohydrates, 2 low fat dairy products, no more than 2 lean meats, as well as 2–3 servings nuts and seeds per week [182]. The DASH diet emphasizes the consumption of “healthy” carbohydrates (e.g., green leafy vegetables, whole grains, legumes) and “good” fats (e.g., foods low in saturated fats, nuts, avocados, fish) [182]. The DASH diet also promotes low sodium intake (<1500 mg/day) and reduction of processed food consumption [183]. Because of these recommendations, the DASH diet has been named an excellent dietary intervention for those with other chronic diseases such as DM, heart disease, and obesity [183].

Chronic disease patients often have impaired GSH statuses and heightened GSSG levels [184]. It has been postulated that the DASH diet may assist in controlling metabolic profiles of those suffering from a chronic disease, in part through its positive impact on GSH status. When the DASH diet was implemented in overweight men and women with NAFLD for 8 weeks without other lifestyle modifications [185], plasma GSH concentrations significantly increased [185]. In addition, plasma total GSH levels increased in overweight and obese women with polycystic ovarian syndrome who adhered to the DASH diet for 8 weeks [186]. Furthermore, pregnant women diagnosed with gestational diabetes at 24- to 28-weeks’ gestation were assigned to either a standard American diet or the DASH diet with 2400 mg sodium per day for 4 weeks [183], and those in the latter group had significantly higher plasma total GSH levels [183]. In conclusion, the DASH diet appears to be a feasible dietary strategy for increasing total GSH levels.

### 4.3. Other Diets

Naturally, vegetarian diets exclude meat, a significant source of amino acids needed for GSH biosynthesis [187]. Yet vegetarian and vegan diets include substantial amounts of fruits and vegetables, which are naturally high in antioxidants [188,189], and these diets are associated with lower rates of obesity, cancer, and diabetes [190]. A vegetarian diet appears to positively influence GSH levels in some populations, such as patients with type 2 diabetes [191] and cardiovascular disease [192]. However, other studies found no significant differences in GSH status between vegetarians and fish or meat eaters [188,193,194]. Yet another study found that a vegan diet with low protein intake results in significantly lower blood GSH levels compared to omnivores and lacto-ovo-vegetarians [187].

A Western-type diet is characterized by high amounts of meats, saturated fat, refined grains, and sugar, with low amounts of fruits and vegetables [195]. The Western diet has been associated with increased risks of cancer and other chronic diseases [179] and is commonly used to experimentally induce oxidative stress and antioxidant depletion [179,196,197,198], including impaired GSH synthesis [197]. Furthermore, Western-type diets are correlated with low vitamin B_6_ [199], methionine [198], and cysteine [198] status, as well as high homocysteine levels [200], suggesting that the diet may affect GSH homeostasis through its effects on precursor availability. Overall, the diet is associated with excessive body fat, insulin resistance, and poor health [201], so an alternative diet emphasizing plants and less processed foods is recommended.

## 5. Other Supplementation Sources

### 5.1. Whey Protein

Whey protein is a rich source of cysteine, so research is being conducted on its relationship with GSH status. Evidence suggests that the consumption of whey protein can raise intracellular GSH levels in healthy individuals [202]. In addition, whey protein intake was found to increase lymphocyte GSH levels in CF patients [203]. More research is needed to evaluate the true effect of whole whey protein supplementation on GSH homeostasis.

### 5.2. Sublingual and Liposomal GSH Supplements

Oral GSH supplementation is not the most effective means of increasing GSH status, so researchers anticipate that alternate forms of oral GSH may be more promising. Notably, food-grade sublingual and liposomal GSH supplements have been designed. Sublingual forms of GSH have received significant attention because sublingual compounds are able to by-pass hepatic first-pass metabolism and degradation, maintaining supplement bioavailability [27]. A 3-week randomized crossover study was conducted on metabolic syndrome patients, where they were supplemented with oral GSH (450 mg/day), oral NAC (200 mg/day), or a sublingual form of GSH (450 mg/day) [27]. Compared to the oral GSH group, sublingual GSH resulted in a significant increase in both total plasma glutathione and plasma GSH [27]. Plasma vitamin E levels also increased in the sublingual GSH group, a result not found in either the oral GSH or NAC supplemented groups [27].

To supplement with lipoceutical GSH, GSH is first packaged into liposomes, small droplets that fuse with cellular membranes, directly releasing their contents into target cells. Recent studies have underscored the beneficial effects of liposomal GSH on intracellular GSH concentrations [204,205]. For instance, liposomal GSH was given to children (3–13 years old) with autism spectrum disorder, and the treatment significantly increased plasma GSH, cysteine, and taurine levels [29].

To date, a limited number of animal and clinical interventions have evaluated the efficacy of liposomal GSH [29,205,206]. Future research is warranted and needs to weigh potential benefits of these pharmaceuticals against their high costs.

## 6. Conclusions

GSH is an exciting and promising therapeutic target against oxidative stress. Given the low bioavailability of oral GSH, researchers have supplemented with compounds that directly or indirectly affect the GSH system. The list of supplements discussed in this review is not exhaustive, and other amino acids and micronutrients, or their combinations, may have significant effects on GSH homeostasis in specific populations. It is important to ensure that interventions are tested in various age groups, in particular because pediatric populations have been consistently understudied. And it is important to consider the health status of each population under investigation, as dysfunction of the liver and other essential organs can impede the body’s ability to synthesize GSH and thus, nutraceutical supplementation may not be as effective as it would be in healthy individuals. Investigators must also consider the analytical method used to quantify GSH and GSSG in each study. GSH and GSSG are often quantified via colorimetric assays [207], high performance liquid chromatography (HPLC) [208], and mass spectrometry [209]. A particular benefit of mass spectrometry and HPLC with electrochemical detection is the direct measurement of GSH without derivatization [208]. Finally, in each study, the source of GSH should be carefully selected. For instance, GSH levels would ideally be measured in serum and RBC, in tandem, to address the fact that GSH may be redistributed from the serum to RBC during times of stress or tissue injury. Overall, supplementation of amino acids and micronutrients, as well as implementation of diet strategies offer safe and non-invasive strategies to improve GSH status and protect the body from oxidative stress in various diseases and conditions.
